# Etymologia: Nipah Virus

**DOI:** 10.3201/eid2505.ET2505

**Published:** 2019-05

**Authors:** Ronnie Henry

**Keywords:** Nipah virus, viruses, Hendra virus, Malaysia, Singapore

## Nipah Virus [neʹ-pə vīʹ-rəs]

In 1994, a newly described virus, initially called equine morbillivirus, killed 13 horses and a trainer in Hendra, a suburb of Brisbane, Australia. The reservoir was subsequently identified as flying foxes (Figure), bats of the genus *Pteropus* (Greek *pteron* [“wing”] + *pous* [“foot”]). In 1999, scientists investigated reports of febrile encephalitis and respiratory illness among workers exposed to pigs in Malaysia and Singapore. (The pigs were believed to have consumed partially eaten fruit discarded by bats.)

**Figure:**
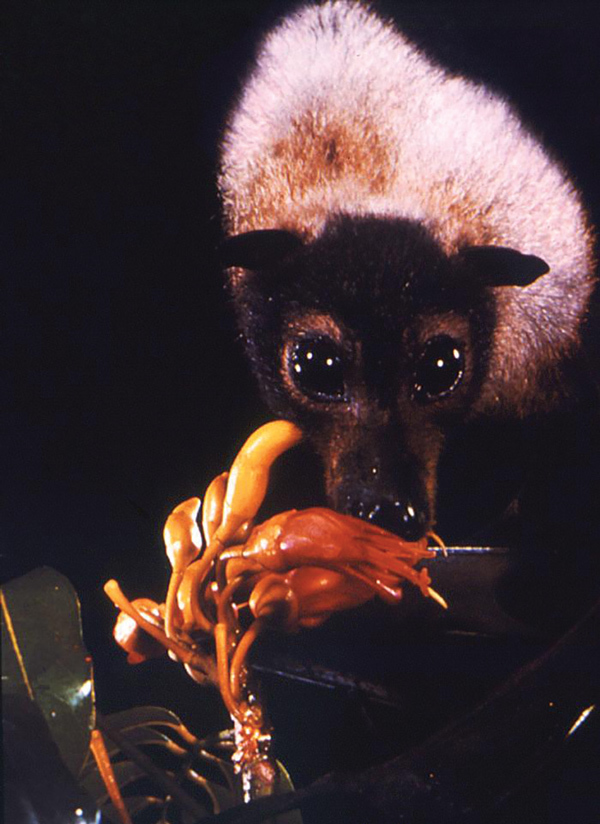
Spectacled flying fox (*Pteropus conspicullatus*) feeding on nectar of unidentified flowers. The natural reservoir for Hendra virus is believed to be flying foxes (bats of the genus *Pteropus*) found in Australia. The natural reservoir for Nipah virus is still unknown, but preliminary data suggest that these bats are also reservoirs for Nipah virus in Malaysia. CDC/Brian W.J. Mahy.

The causative agent was determined to be closely related to Hendra virus and was later named for the Malaysian village of Kampung Sungai Nipah. The 2 viruses were combined into the genus *Henipavirus*, in the family *Paramyxoviridae*. Three additional species of *Henipavirus*—Cedar virus, Ghanaian bat virus, and Mojiang virus—have since been described, but none is known to cause human disease. Outbreaks of Nipah virus occur almost annually in India and Bangladesh, but *Pteropus* bats can be found throughout the tropics and subtropics, and henipaviruses have been isolated from them in Central and South America, Asia, Oceania, and East Africa.
